# Multimodal imaging of congenital pyriform fossa fistula in children

**DOI:** 10.3389/fped.2023.1089241

**Published:** 2023-03-21

**Authors:** Aiguo Zhai, Xuehua Peng, Yu Guo, Jian Li, Jianbo Shao

**Affiliations:** Department of Imaging Center, Wuhan Children's Hospital (Wuhan Maternal and Child Healthcare Hospital), Tongji Medical College, Huazhong University of Science and Technology, Wuhan, China

**Keywords:** children, pyriform fossa fistula, thyroid gland, multimodal imaging, infection

## Abstract

**Objective:**

Our aim was to explore the clinical value of multimodal imaging examinations in the diagnosis of congenital pyriform fossa fistula in children, so as to provide clues for the early diagnosis and treatment of congenital pyriform fossa fistula.

**Methods:**

The clinical and imaging data of 55 children with pyriform fossa fistula diagnosed surgically in our hospital from 2015 to 2018 were analyzed retrospectively. All 55 patients underwent a CT scan. Of those patients, contrast enhancement CT was performed in 47 cases, MRI was performed in 2 cases, and barium esophagography was performed in 41 cases.

**Results:**

Among the 55 cases, there were 24 male patients and 31 female patients. The age ranged from 11 months to 13 years old, and the median age was 3.8 years old. The lesions of 49 cases (89.1%) were located on the left side, and the imaging of CT showed soft tissue mass in the anterior cervical region blurred boundary. There was ipsilateral thyroid involvement in 50 cases (90.9%), trachea and/or carotid sheath extension in 43 cases (78.2%), abscess formation in 39 cases (70.9%), and pneumatosis in 25 cases (45.5%). The CT examination of 22 children after treatment showed a linear or tubular low-density shadow in the thyroid gland, gas accumulation in the anterior cervical region or thyroid, and residual contrast medium, partly. A total of 24 cases underwent barium esophagography during the acute phase, and 15 cases (62.5%) showed sinus formation from the pyriform fossa downward or punctate high-density shadow in the anterior cervical region. The 2 cases where MRI was performed showed abscess formation in one side of the neck and thyroid involvement.

**Conclusion:**

Pyriform fossa fistula is most common in the left anterior cervical region, and it is closely related to the thyroid gland. The plain and enhanced-contrast CT scan can be used as the first choice during the infection stage. It helps to understand the location, extent, and structure of the surrounding tissue. The preliminary diagnosis of pyriform sinus fistula was according to the imaging features. It provided an important basis for clinical diagnosis and reduced the pain caused by repeated infection or surgical incision and drainage.

## Introduction

Congenital pyriform fossa fistula (CPSF) is a rare cervical branchial malformation. The pyriform fossa originates from the third and fourth branchial sacs during the embryonic stage. CPSF is caused by a sinus connected to the pyriform fossa from the unclosed pharyngeal sac, perforated branchial membrane, or unclosed cervical sinus (1, 2). It often occurs in children. It presents as a neck mass in acute infection with fever, pharynx discomfort, and other symptoms. In most cases, there is only an internal fistula without an external fistula. There are no obvious clinical symptoms and signs during the non-infectious period; in addition, some clinicians have no experience with this disease. Even during infection, it is often misdiagnosed as common inflammation with abscess formation. Most pediatric patients suffer from long-term repeated infection or multiple abscess incisions and drainage because of delay in diagnosis. Because of the limitations of the scanning area, the non-speciﬁc presentation, and the less-experienced operator, the accuracy of ultrasound (US) is lower than that of CT and MRI (3, 4). The study summarized the imaging manifestations of CT, barium esophagography, and MRI of pediatric CPSF in different periods, so as to provide the reference for clinical diagnosis and treatment of pediatric CPSF as soon as possible.

## Materials and methods

### Patients

The clinical and imaging data of 55 children with CPSF confirmed by surgery in Wuhan Children's Hospital were collected from January 2015 to December 2018, including 24 male patients and 31 female patients. The age of initial diagnosis was from 11 months to 13 years old, and the median age was 3.8 years old. All of them went to the hospital because of acute cervical mass, of which 35 cases had a history of repeated infection, incision, and drainage of neck abscess.

### Computed tomography protocols and evaluation

Siemens SOMATOM Definition AS128 CT scanner with a low dose was used to scan the neck. From the oropharynx to the sternoclavicular joint level, slice thickness was 5.0 mm and slice spacing was 5.0 mm, and enhanced contrast agent iohexol at a dosage of 1.2–1.8 ml/kg and flow rate of 0.4–1.2 ml/s was used. After scanning, a thin slice of 1.0 mm reconstruction and multi-directional MPR reconstruction was performed. The patients were sedated with oral chloral hydrate according to the 0.5 ml/kg standard (the maximum is not more than 10 ml). After sedation, the pediatric patients were scanned by CT.

### Barium esophagography

The examinations were obtained with a digital gastrointestinal radiography (SONIALVISIONG4, Shimadzu, Japan). Iodine contrast agent (Iohexol Injection, 350 mg I/ml) was an oral contrast agent used for pediatric patients younger than 1 year old, and the maximum dose was 20 ml. Barium dilution was used for patients older than 1 year old, and the maximum dose was 40 ml. Images were performed in the positive position, left anterior oblique position, right anterior oblique position, and lateral position, respectively.

### Magnetic resonance imaging protocols and evaluation

Images were acquired at a 3.0 T (Discovery MR750, GE, USA) using an 8-channel head and neck coil. The scan sequence includes SE T1WI, FSE T2WI, and FIR STIR. The thickness of layers is 3.5 mm, the distance between layers is 1 mm, the FOV is 24 cm × 24 cm, and the matrix is 512 cm × 512 mm. The contrast medium of the enhanced scan was Gd-DTPA, and the dose was 0.2 mmol/kg. Cross-sectional and coronal scans were performed after the injection of the contrast agent.

## Results

**Figure 1:**
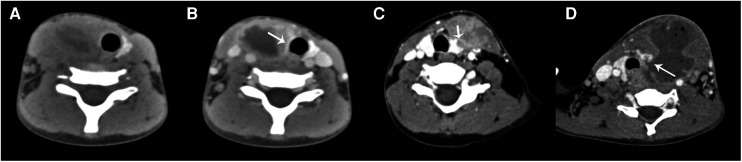
(**A,B**) A 4-year-old girl had a right neck mass for 5 days. (**A**) CT plain scan showed a soft tissue mass in front of the right neck with necrotic lesions. (**B**) Contrast-enhanced CT showed abscess wall thickening and annular enhancement; the right lobe of the thyroid presented a low-density shadow and unclear boundary (arrow). The trachea and carotid sheath were displaced. (**C**) A 6-year-old boy had a left neck mass for 14 days. Contrast-enhanced CT showed a soft tissue mass in the front of the left neck with uneven enhancement; the left lobe of the thyroid was involved with the low-density shadow extending to the mass (arrow). (**D**) A 9-year-old boy had a left neck mass with a fever for 4 days. Contrast-enhanced CT showed the formation of a left anterior cervical abscess, accompanying gas accumulation, displacement of trachea and carotid sheath, and stenosis of the left internal jugular vein; the left lobe of the thyroid and the posterior pharyngeal wall were involved with the low-density shadow extending to the abscess (arrow).

**Figure 2:**
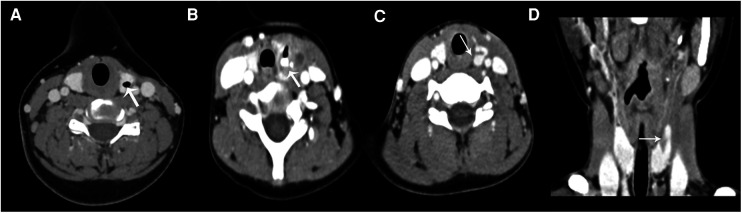
(**A**) A 12-year-old boy had a left neck mass for 8 years. Contrast-enhanced CT showed the local gas accumulation in the left lobe of the thyroid gland (arrow). (**B**) A 4-year-old boy had a recurrent infection in the left neck for more than 20 days. The thyroid showed local gas accumulation and high-density contrast medium residue (arrow); adjacent soft tissue swelling showed a low-density shadow. (**C**) (axial) and (**D**) (coronal). A 7-year-old boy had a left neck mass for 8 months. The contrast-enhanced CT showed a linear low-density shadow in the upper pole of the left lobe of the thyroid gland (arrow).

CT was performed in all 55 cases, including 8 cases of CT plain scan, 47 cases of plain and contrast-enhanced scan, 2 cases of plain and contrast-enhanced MRI scan, and 41 cases of barium esophagography in 55 cases. All CPSF occurred in one side of the anterior cervical triangle: 49 cases on the left side (89.1%) and 6 cases on the right side. The main manifestations of CPSF were soft tissue mass between the medial sternocleidomastoid muscle and thyroid; the central density was decreased, the periphery was unclear, and the boundary was blurred. In some cases, the swelling density of the subcutaneous fat layer was increased, and the range was about C3–6 vertebral plane. Due to the formation of abscess, 39 cases (70.9%) showed mild inhomogeneous enhancement; the abscess wall showed thick-walled circular enhancement and non-enhancement in the central liquefied necrotic area (Figures 1A,B). 50 cases (90.9%) were associated with ipsilateral thyroid involvement showing localized patchy low-density areas in the thyroid (Figure 1C), and a little gas accumulation could be seen in the abscess cavity in 25 cases (Figure 1D). The trachea and/or ipsilateral carotid sheath moved outwards and posteriorly in 43 cases (78.2%), internal jugular vein narrowed in 15 cases (27.3%), obvious swelling and thickening and uneven enhancement in ipsilateral sternocleidomastoid muscle could be seen in 38 cases (69.1%), and swelling and shallower soft tissue in one side of the Pyriform fossa could be seen in 26 cases (47.3%). In 23 cases (41.8%), CPSF was accompanied with slightly larger cervical lymph nodes; the maximum diameter was less than 20 mm, the enhancement was uniform, and no fusion was found. The lesion extended backward to the anterior space of the posterior pharyngeal wall in 5 cases (9.1%). Of the 55 cases, 22 cases (50%) showed low-density gas accumulation in one side of the neck or thyroid lobe after treatment (Figure 2A), 7 cases (31.8%) showed contrast medium residue (Figure 2B), and 14 cases (63.6%) showed line-like or tubular low-density shadow above the left/right lobe of the thyroid (Figures 2C,D).

**Figure 3:**
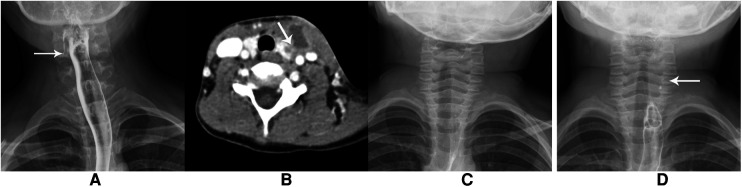
(**A**) A 10-year-old girl had a recurrent infection in the right neck for 8 years. The barium esophagography showed the strip high-density shadow on the right side, indicating pyriform fossa fistula. (**B–D**) A boy aged 4 years and 10 months old had a recurrent mass in the left neck for 3 years. (**B**) contrast-enhanced CT showed the formation of an abscess in front of the left neck and involvement of the left lobe of the thyroid. (**C**) The barium esophagography showed normality 3 days after infection. (**D**) The esophagography showed punctate contrast agent residue in the left neck one month later, suggesting pyriform fossa fistula.

In total, 41 patients underwent barium esophagography with a median age of 2 years, of which 24 cases were examined in the acute phase; 15 cases (approximately 62.5%) indicated the formation of sinus below the pyriform fossa on one side (Figure 3A), and 9 cases (37.5%) showed no obvious abnormality (Figures 3B,C). The reexamination showed the existence of pyriform fossa fistula (Figure 3D). The examination was terminated in 1 case because the child did not cooperate.

The interval between the last symptom before operation and examination was calculated. 34 cases underwent only one barium esophagography examination with a median time of 25 days. 31 cases (75.6%) showed the formation of sinus below the pyriform fossa on one side (Figure 3A). The range was approximately 8 mm ∼50 mm, and 3 cases did not show the sinus. In 7 cases, the sinus did not display in the first examination with a median time of 4 days and displayed with a median time of 43 days in the second examination.

**Figure 4:**
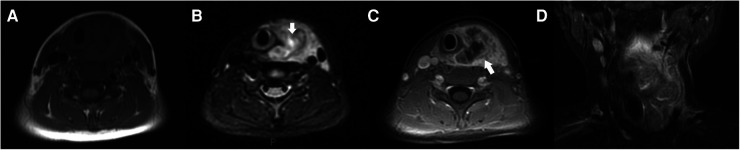
(**A–D**) A boy aged 8 years and 10 months had a left neck mass with a fever for 5 days. (**A**) T1WI and (**B**) T2WI STAIR: The mass in the left anterior region of the neck showed iso-and hypo-intensity on T1WI and hyper-intensity on T2WI with the unclear boundary and the necrotic area (arrow); the adjacent trachea and carotid sheath are pushed, and the left lobe of the thyroid is not clearly displayed. (**C**) The contrast-enhanced MRI showed inhomogeneous thick-walled enhancement of the mass in the left anterior cervical region (arrow) with the abscess and involvement of the left lobe of the thyroid. (**D**) The coronal contrast-enhanced MRI distinctly showed the extent of lesion involvement.

MRI examination in 2 cases showed soft tissue mass in the left anterior cervical region with blurred boundary, low signal on T1WI and high signal on T2WI in the central necrotic area, and displacement of the adjacent trachea and large vessels. The ipsilateral thyroid is not clear (Figures 4A,B). After contrast enhancement, irregular thick-walled enhancement with ipsilateral thyroid involvement can be seen (Figures 4C,D).

## Discussion

CPSF is caused by the incomplete degeneration of the third or fourth pharyngeal sac, also known as the third or fourth branchial fistula, during early embryonic development. It often occurs in childhood, and there is no significant gender difference. More than 90% of the cases are located on the left side of the neck (5–9). 89.1% of the cases in this group are located on the left side, which is consistent with the reports in previous works of literature. The pyriform fossa fistula originating from the third branchial sac begins at the bottom of the pyriform fossa, goes above the superior laryngeal nerve, surrounds the hypoglossal nerve, and descends to one side of the neck. The pyriform fossa fistula originating from the fourth branchial sac begins at the tip of the pyriform fossa, runs obliquely at the inferior lateral side of the thyroid cartilage, and terminates at the thyroid or continues to course through the thyroid tissue and descends to the back of the ipsilateral sternoclavicular joint (5, 10, 11).

There was no obvious external fistula on the surface of the skin of most children with CPSF. 55 children in this group had no external fistula at the first onset and no obvious clinical symptoms and signs during the non-infectious period. Most of them were diagnosed with fistula inflammation caused by upper respiratory tract infection mainly presenting as a mass in one side of the anterior cervical triangle with redness and swelling, pain, dysphagia, fever, and so on. A purulent cavity can be formed in a short time, and a few can rupture by themselves. CPSF is relatively rare in clinical practice. Because of the lack of understanding of its special embryonic developmental process and complex local anatomical structure (12), it is easy to be misdiagnosed as a general deep neck abscess or suppurative thyroiditis; hence, only anti-inflammatory treatment or abscess incision and drainage is given. Even thyroidectomy was performed due to misdiagnosis before operation. After the disappearance of clinical symptoms, the children were discharged from the hospital and often went to see a doctor repeatedly because of recurrent infections. In this group, there were 35 patients suffering from recurrent neck infections; 29 cases (29/55, 52.7%) had undergone abscess incision and drainage many times, of which the longest medical history was about 8 years.

Therefore, CPSF should be highly suspected when there is a painful mass in one side of the anterior cervical triangle, especially when there is a history of repeated infection and abscess in the left neck.

The direct signs of CPSF during the acute stage were soft tissue mass between the medial side of the sternocleidomastoid muscle and thyroid gland, blurred boundary, swelling of the ipsilateral sternocleidomastoid muscle, and flocculent shadow of the subcutaneous fat layer in the neck. The central lower-density area and gas accumulation appeared in abscess formation. After enhancement, there was uneven enhancement, no enhancement in the central necrotic area, and irregular ring enhancement in the abscess wall. Abscess formation was seen in approximately 70.9% of the cases, of which approximately 60% showed gas accumulation or abscess in the thyroid gland. A study reported that more than 50% of patients show an air-ﬁlled cystic mass on CT or MRI scans (5). The air within the thyroid gland was diagnostic, which indicated the presence of a potential sinus tract, especially for patients without thyroid abscesses. It was detected in 19 (16.5%) of our patients. The sinus tract crossing the thyroid gland was found in 38 (33%) of our patients (4). The adjacent tissues and organs were displaced. Approximately 78.2% of the patients in this group showed varying degrees of tracheal displacement to the healthy side and/or ipsilateral carotid sheath to the lateral and posterior area. In addition, CT showed soft tissue swelling of the pyriform fossa, shallowness of the pyriform fossa, narrowing of the ipsilateral internal jugular vein, and so on. The most important indirect signs were associated with ipsilateral thyroid involvement in most cases, which showed irregular patchy low-density shadows in the thyroid gland. Thyroid involvement accounted for approximately 90.9% of this group of cases. The cause was considered to be related to the course of the fistula. The pyriform fossa fistula terminates in the upper polar of the thyroid gland or passes through the upper polar of the thyroid gland to the deep cervical fascia (13). On MRI, T1WI showed iso- or slightly hypo-signal intensity, and T2WI showed slightly high-signal intensity and inhomogeneous enhancement with ipsilateral thyroid involvement after enhancement. The hyperintensity of the lesion on T2WI and DWI indicated the formation of an abscess. It showed the thick wall and irregular ring enhancement after contrast. MRI has high soft-tissue resolution, permits multi-sequences and multi-angles imaging, and easily shows the anatomic structure of soft tissue. MRI reports of CPSF showed that a tunnel-like lesion between the pyriform fossa and the upper pole of the thyroid gland was detected in 46 (40%) patients. This report indicated the existence of the sinus tract, delineated the pathway of the sinus tract, and possibly displayed the location of the internal ﬁstula opening, which was a key point in differentiating between the third and fourth pouch anomalies (4).

A few studies showed that pyriform sinus fistula was detected when the barium swallow examination was performed after inflammation had subsided. The findings from our study indicated that the timing of the examination and barium concentration to detect the fistula is important (12, 14). Among the 133 children with a history of the ﬁrst esophagography timing, the ﬁstulous tract was identiﬁed in 115 children (86.47%) using the ﬁrst esophagography (12). In our studies, the true-positive rate of the ﬁrst esophagography was 75.6%. The timing of the ﬁrst esophagography with a true-positive result was signiﬁcantly longer than that of the examination with a false-negative result. A recent study showed joint examination of barium esophagography, and an immediate CT is the preferred imaging modality for the diagnosis of CPSF in children (12). The non-compliance of children, the risk of aspiration, and the high false-negative results should also be considered.

We should give more attention to the differential diagnosis. (1) Primary suppurative thyroiditis: because most of the CPSF passes through or terminates in the thyroid, during the infection stage, abnormal changes appear in the shape, size, and density of the ipsilateral thyroid, which is similar to that of primary acute thyroiditis. However, the thyroid is not susceptible to bacteria that cause suppurative infection because of the integrity of the thyroid capsule, abundant blood supply, lymphatic reflux, and high iodine concentration in the thyroid (15). Takai first reported that suppurative thyroiditis was related to congenital pyriform fossa fistula (16), and other scholars have found that pyriform fossa fistula is the main cause of acute thyroiditis in children, especially in recurrent cases (17). Nearly 90.9% of the patients in this group showed ipsilateral thyroid involvement. (2) Acute necrotizing lymphadenitis: it is mainly manifested as multiple enlarged lymph nodes in the unilateral or bilateral submandibular or posterior cervical triangle, relatively less in the anterior cervical triangle, and less thyroid involvement. In this group, the reactive lymph nodes near the lesions were enlarged in 23 cases with homogeneous enhancement. (3) Thyrohyoid cysts: they were often located in the anterior midline of the neck between the thyroid cartilage and hyoid bone. A few of them were located in the thyroid isthmus which is different from CPSF (which often involved the lateral lobe of the thyroid). An enhanced CT scan showed no enhancement or cyst wall enhancement. Anterior cervical round or quasi-round low-density mass with no redness, swelling, and tenderness could still be found in the non-infectious stage, while CPSF in the non-infectious stage could be negative and misdiagnosed. (4) The first or second branchial cleft anomaly: the lesion of the first branchial cleft anomaly occurred in the Pochet's trigonum, being closely related to the external auditory canal and the parotid gland (18). The external opening is mostly located around the ear or above the level of the submandibular hyoid, and the internal opening can be located in the external auditory canal. The lesion of the second branchial cleft anomaly is located in the carotid triangle, also extending to the parapharyngeal space. The external opening is mostly located in the mid or lower third of the anterior border of the sternocleidomastoid muscle, and the internal opening is rarely located at the tonsillar fossa, often presenting a sinus with variable extension into the neck (19, 20). The lesion can cross the whole cervical side, and it is located between the left lobe of the thyroid and the sternocleidomastoid muscle and can also affect the left lobe of the thyroid. It is necessary to distinguish them accurately from each other by the clinical history and the characteristic imaging findings.

Ultrasonography is the first choice for the cervical mass, which has the advantages of being dynamic, simple, non-invasive, and non-radiation, but compared with the tissue resolution and post-processing function of CT and MRI images, ultrasound often does not show clearly the relationship between the lesion and the surrounding tissue and the source of cervical infection. It is difficult to distinguish from other inflammatory lesions of the neck. Barium esophagography is relatively fast and simple, and children do not need to be sedated. It can directly show the formation of pyriform sinus and make a clear diagnosis, but there are some limitations in hypopharyngeal radiography during acute infection. These are mainly due to local soft tissue edema and mucosal thickening during acute inflammation, and it is often not easy to find the fistula. There is also the limitation of the relatively young age of some children coupled with neck pain and discomfort. Children cannot cooperate well during the examination process resulting in the interruption of the test or having false negative results, so it is generally recommended that the examination should be carried out after the inflammation has subsided. MRI examination has no radiation, has good soft tissue resolution, and can clearly show the characteristics of the lesions and the relationship between adjacent tissues. However, the time of MRI examination is relatively long, the noise is loud, children do not cooperate without being sedated, and the clinical application is not common. The circular internal opening of the mucous membrane at the basolateral wall and the tip of the pyriform fossa can be displayed directly under the electronic nasolaryngoscope. It is the gold standard for the diagnosis of congenital pyriform fistula (21, 22).

However, when the child is in the acute infection stage, the mucosal swelling can cover the internal opening, and the flexible electronic nasolaryngoscope can't stretch the mucosa at the pyriform fossa, resulting in a false negative.

Therefore, when a child has a painful mass in one side of the anterior cervical triangle, or a history of repeated infection or abscess on one side (especially the left side), according to the direct or indirect results of CT or MRI although not all the fistulas can be directly displayed, we should be highly alert to the possibility of pyriform fossa fistula (CPSF). Plain and enhanced CT or MRI scan is helpful to observe the occurrent site of the mass and the morphology, the structure, and the location of its adjacent tissue. At the same time, the patients can be evaluated before the operation and followed up after the operation. The method of barium esophagography or CT examination after esophagography in the non-infectious period can further confirm the diagnosis of CPSF and improve the coincidence rate of diagnosis (23). Appropriate imaging examinations are selected according to different periods to provide a basis for further clinical diagnosis and treatment.

## Data Availability

The original contributions presented in the study are included in the article/Supplementary material, further inquiries can be directed to the corresponding author/s.
